# Supplementary material for: Mechanistic insights into steroid hormone-mediated regulation of the androgen receptor gene
Source: PLoS One. 2024 Aug 1;19(8):e0304183. doi: 10.1371/journal.pone.0304183 (PMC11293711; doi:10.1371/journal.pone.0304183)
Supplement: S1 Fig — (A) Images of Western Blots for listed steroid hormone receptors in different prostate cell lines. β-Actin shown as a housekeeping gene and loading control. (B) Table summary of Western Blot data; +, expression, +/-, modest expression; and -, negative. (PDF) [file pone.0304183.s001.pdf]

A

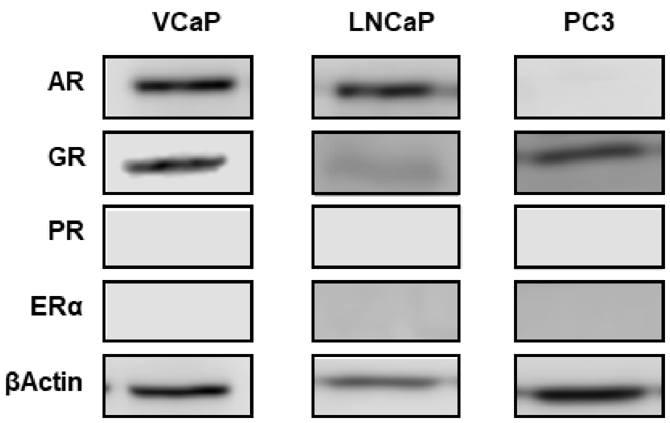

B

**Receptor**	VCaP	LNCaP	PC-3
Androgen Receptor	+	+	-
Glucocorticoid Receptor	+	-/+	+
Progesterone Receptor	-	-	-
Oestrogen Receptor α	-	-	-

S1 Fig. Steroid hormone receptors in prostate cancer cell lines.
