# Supplementary figures and images for: Mechanistic insights into steroid hormone-mediated regulation of the androgen receptor gene
Source: PLoS One. 2024 Aug 1;19(8):e0304183. doi: 10.1371/journal.pone.0304183 (PMC11293711; doi:10.1371/journal.pone.0304183)

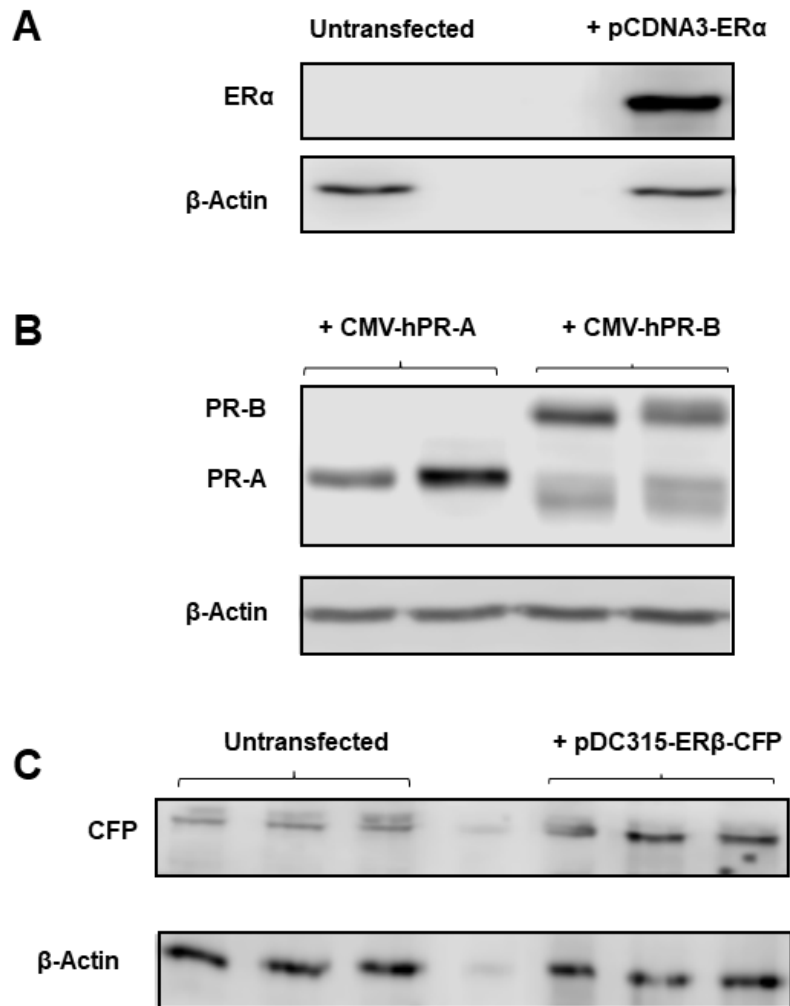

**S2 Fig. Transfection with oestrogen and progesterone receptors.**

Supplement: S2 Fig — (A) Anti-ERα immunoblots of protein extracts taken from VCaP cells in the presence or absence of pCDNA3-ERα transfection. (B) Anti-PR immunoblots of protein extracts taken from VCaP cells transfected with CMV-hPR-A and CMV-hPR-B. The PR antibody used detects both PR isoforms. (C) Anti-CFP immunoblots of protein extracts taken from VCaP cells in the presence or absence of pDC315-ERβ-CFP. β-Actin shown as a loading control in all cases. Note a band occurs in the untransfected cells with the αCFP antibody, suggesting cross-reactivity with cellular proteins. (PDF) [file pone.0304183.s002.pdf]

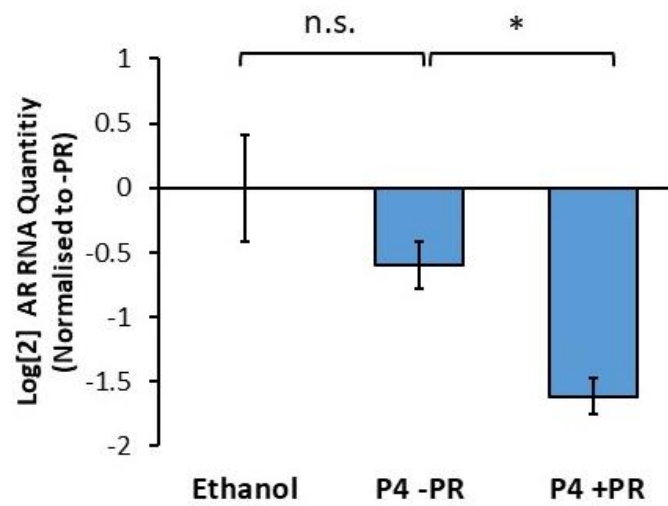

**S4 Fig. Progesterone only affects AR transcription via the activated progesterone receptor.**

Supplement: S4 Fig — qRT-PCR of RNA samples harvested from VCaP cells treated with vehicle (Ethanol) or 10 nM progesterone (P4) for 24 hours, in the absence (-PR) or presence of hPRB (+ PR); GAPDH used as an endogenous control for data normalisation. Error bars show ± standard error of the mean (3 biological replicates). * = p < 0.05 (Unpaired Student’s t-test). (PDF) [file pone.0304183.s004.pdf]

AR Primer Alignment

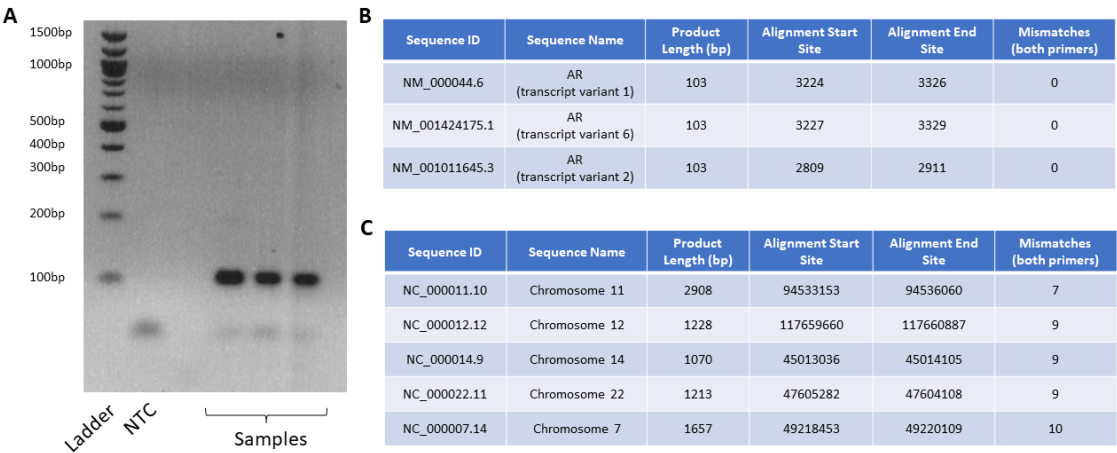

**S5 Fig. Androgen receptor primers are specific to the AR transcript.**

Supplement: S5 Fig — (A) PCR products from AR amplification were run on a 2% agarose gel along with a non-target control (NTC) sample and a 100 bp DNA Ladder (New England Biolabs). Ladder band sizes are marked. (B and C) NCBI Primer BLAST results against all Refseq Homo sapiens mRNA and Refseq Homo sapiens genome sequences respectively. These tables show every sequence to which primers align with <5 mismatches per primer. (PDF) [file pone.0304183.s005.pdf]

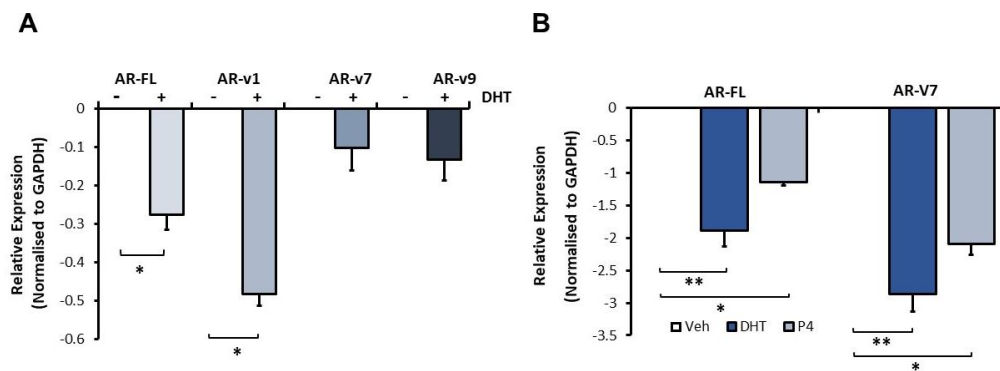

**S6 Fig. Down-regulation of full-length and receptor splice variant mRNA.**

Supplement: S6 Fig — (A) In 22Rv1 cells, transcripts containing cryptic exons (CEs) 1, 3 and 5 were all detected, albeit to differing levels. To interrogate the effects of androgen signalling on AR-v levels we compared transcript levels in cells treated with either vehicle or 10 nM hormone for 24 hours by qRT-PCR. (B) In VCaP cells, it was possible to treat with both DHT and, in the presence of PR-B, progesterone. Consistent with earlier experiments, we noted repression of AR-FL transcripts in the presence of either hormone (A and B). The same was true of AR-V1 (22rv1 cells) and AR-V7 (VCaP cells) transcript levels when compared to vehicle control. Treatment with either DHT (p = 0.009) or progesterone (p = 0.017) resulted in a significant reduction in expression of CE3-containing transcripts. (PDF) [file pone.0304183.s006.pdf]

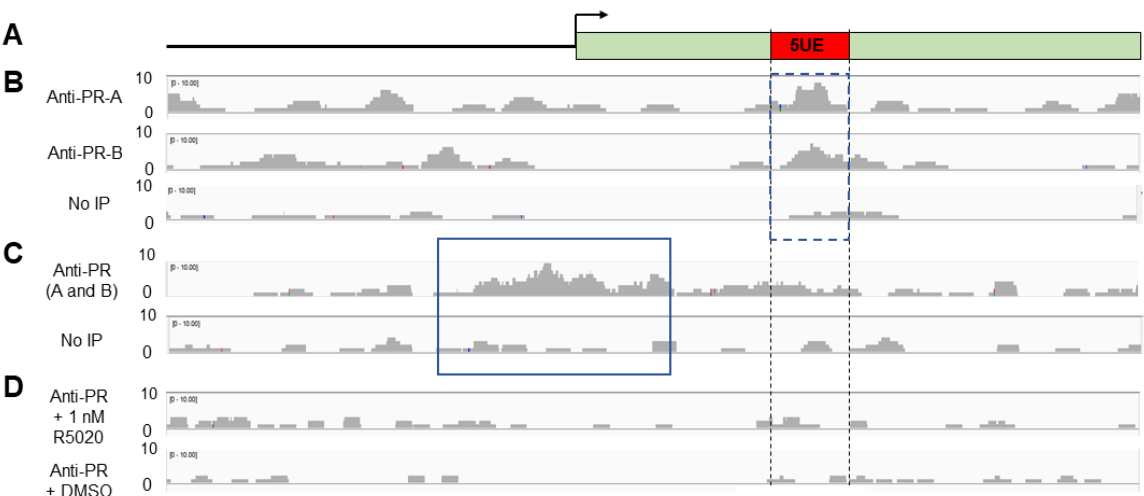

**S8 Fig. Hormone receptor binding to the AR 5'UTR in other tissue types.**

Supplement: S8 Fig — Publicly available ChIP-seq datasets were used to interrogate binding of progesterone receptor to the AR 5’UTR. A 3 kb window is shown, surrounding the putative 5’ UTR receptor binding element (5UE), which is located between the dotted lines. Coverage of this region is shown for each experiment, with peak height corresponding to read depth (see scale on left of each bar). In all cases, peaks which differ significantly between conditions, as identified by MACS2, are indicated by blue box. (A) shows the genomic region of interest (hg19 chrX:66762600–66765599) to provide scale for ChIP-seq interpretation and the 5’UTR (5UE) region (chrX:66764465–66764686) is highlighted. Below this, PR data sets are explored. These include, (B) normal endometrial stroma, with ChIP-seq data from either PR-A (SRR1614984); PR-B (SRR1614985) or no IP (SRR1614984) datasets. (C) Leiomyoma (endometrial tumour) patient samples, from either anti-PR (SRR10189640) or no IP (SRR10189645) datasets. (D) Breast cancer cell line samples, with ChIP-seq against PR in the presence of 1 nM R5020 (SRR15064561) or DMSO vehicle (SRR15064553). (PDF) [file pone.0304183.s008.pdf]

2  
3  
4  
5  
6  
7  
8  
9  
10  
11  
12  
13  
14  
15  
16  
17

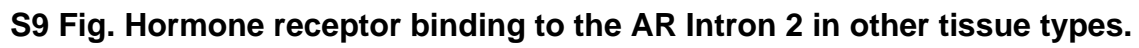

Supplement: S9 Fig — Publicly available ChIP-seq datasets were used to interrogate binding of the progesterone receptor to the AR Intron 2. A 3 kb window is shown, surrounding the putative Intron 2 receptor binding element (I2E), which is located between the dotted lines. Coverage of this region is shown for each experiment, with peak height corresponding to read depth (see scale on left of each bar). In all cases, peaks which differ significantly between conditions, as identified by MACS2, are indicated by blue boxes, with the dotted lines indicating the 12E region of interest. (A) Shows the genomic region of interest (hg19 chrX:66865500–66868499) to provide scale for ChIP-seq interpretation. The intron 2 region (I2E) (chrX:66866941–66867339) is highlighted. Below this, PR experiments are explored. These include, (B) normal endometrial stroma, with ChIP-seq data from either PR-A (SRR1614984); PR-B (SRR1614985) or no IP (SRR1614984) datasets. (C) Leiomyoma (endometrial tumour) patient samples, from either anti-PR (SRR10189640) or no IP (SRR10189645) datasets. (D) Breast cancer cell line samples, with ChIP-seq against PR in the presence of 1 nM R5020 (SRR15064561) or DMSO vehicle (SRR15064553). (PDF) [file pone.0304183.s009.pdf]

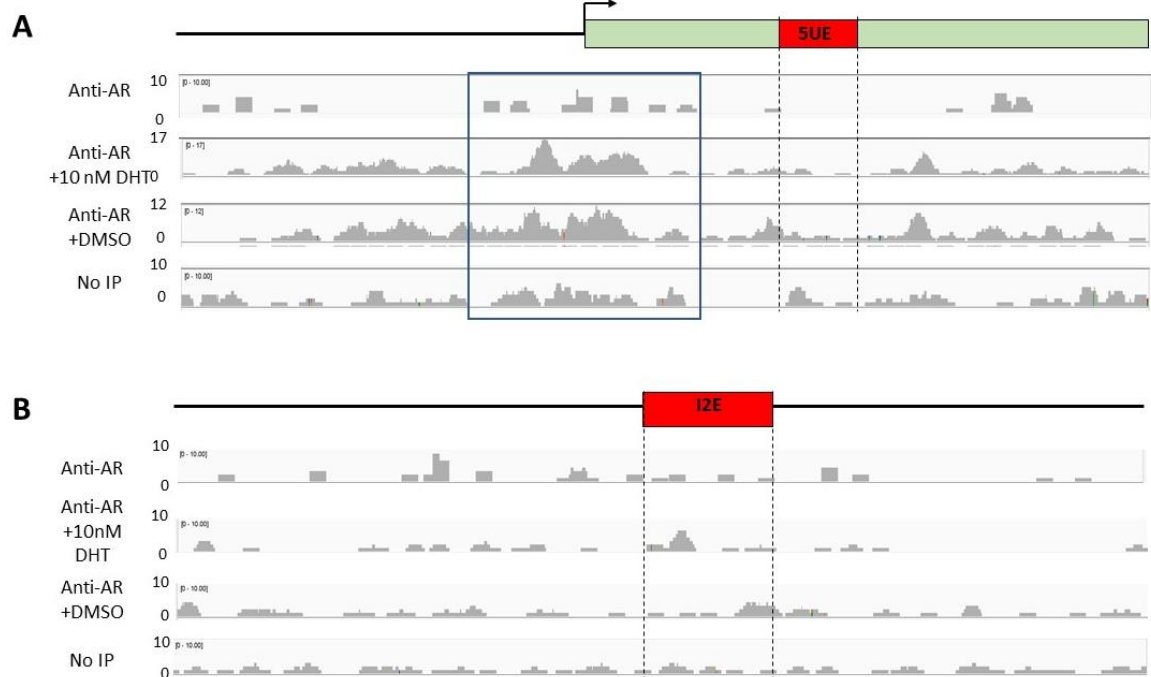

**S10 Fig. Androgen receptor binding to the AR 5'UTR and Intron 2 regions in other tissue types.**

Supplement: S10 Fig — (A) Publicly available ChIP-seq datasets were used to interrogate AR binding to the 5’UTR (A) and intron 2 sequences of the AR gene (B). Peaks which differ significantly between conditions, as identified by MACS2, are indicated by solid blue box. For the AR we analysed PCOS Patient endometrial stroma (SRR7782796), AR ChIP-seq only, and Breast Cancer cell line samples. AR ChIP-seq data is shown from cells treated with 10 nM DHT (SRR12626838) and DMSO vehicle (SRR12626837). A no-IP input sample is also shown for comparison (SRR12626843). We observed little evidence of AR binding at the 5’ UTR element in endometrial stroma (Part A, Top Line). However, in data derived from breast cancer cells, we found a peak which was significantly enriched in anti-AR ChIP compared to no immunoprecipitation controls, suggesting that AR binds proximal to the transcriptional start site of the AR gene. (Part A, Lines 2 to 4). This peak did not overlap with the putative 5’ UTR element we found enriched in prostate cells in response to hormone treatment. No evidence of AR binding to the I2E region was observed in either endometrial stromal samples or breast cancer cells (Part B). (PDF) [file pone.0304183.s010.pdf]

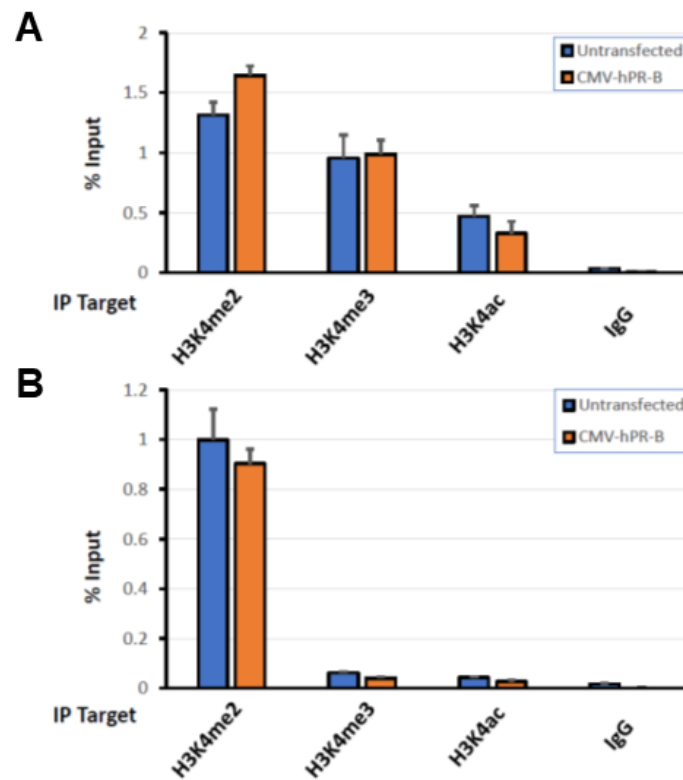

**S11 Fig. Expression of progesterone receptor B does not affect histone marks at the *AR* gene.**

Supplement: S11 Fig — VCaP cells were either transfected with CMV-hPR-B or remained untransfected. 24 hours later, chromatin was harvested, fragmented by sonication, and immunoprecipitated with the appropriate antibody (IgG used as a non-binding isotype control). Precipitated DNA quantified via qPCR using primers for (A) the 5’ UTR Element or (B) the intron 2 Element. Error bars show standard error of the mean (3 biological replicates). (PDF) [file pone.0304183.s011.pdf]

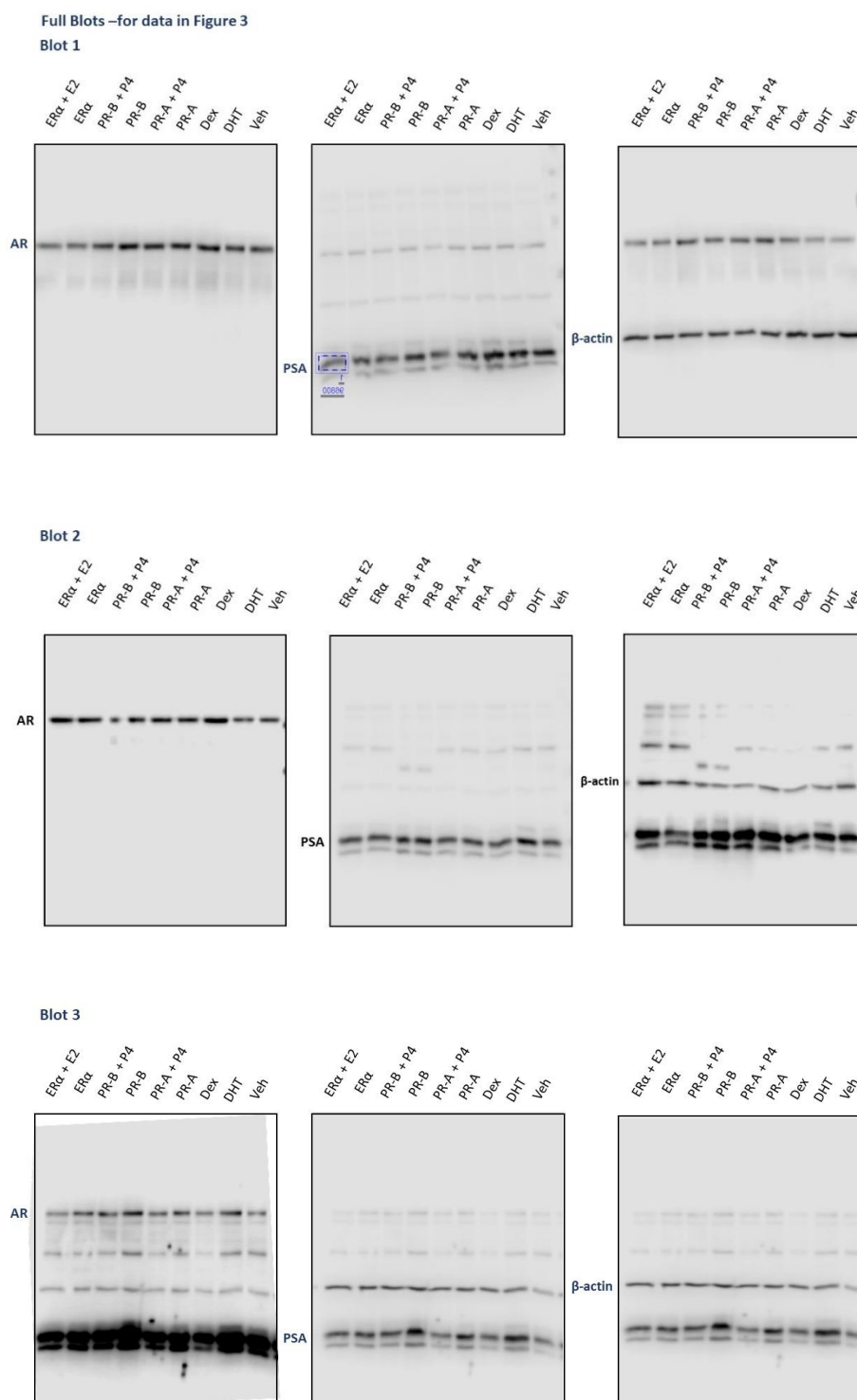

**S13 Fig. Uncropped blots for data presented in Fig 2.**

Supplement: S13 Fig — (PDF) [file pone.0304183.s013.pdf]

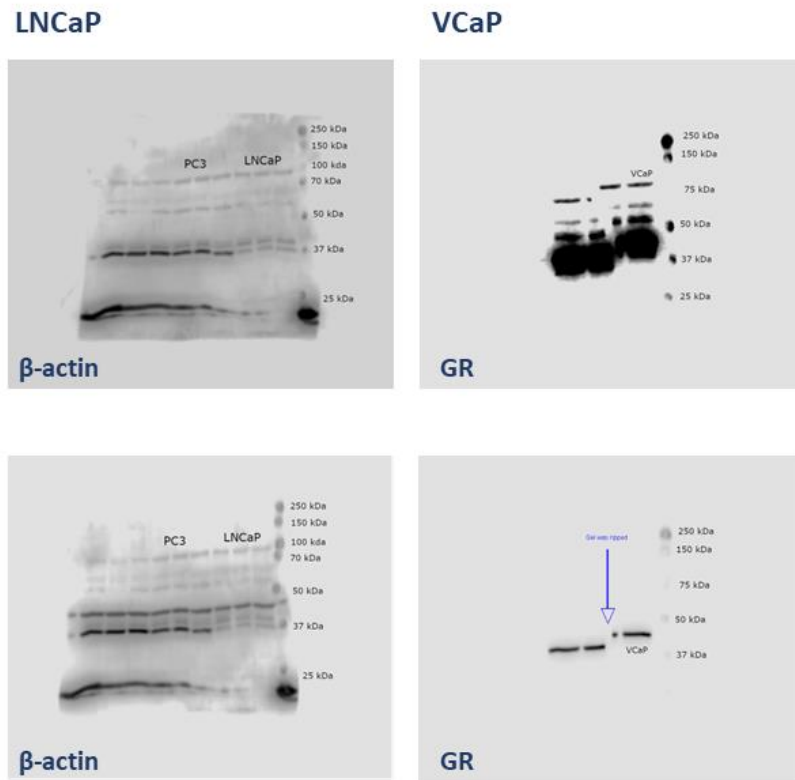

**S14 Fig. Uncropped blots for data presented in Supplementary S1 Fig.**

Supplement: S14 Fig — (PDF) [file pone.0304183.s014.pdf]

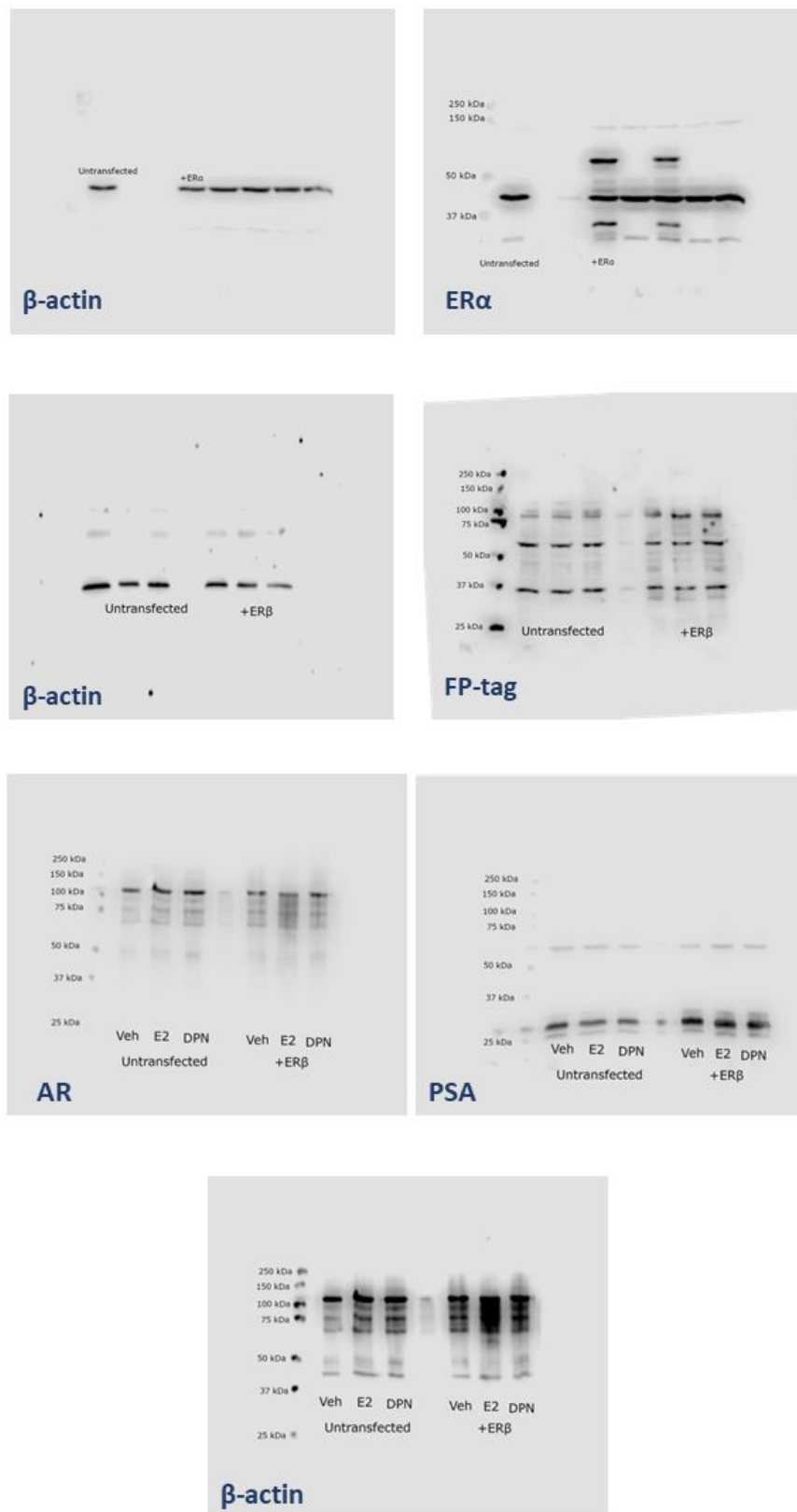

**S15 Fig. Uncropped blots for data presented in Supplementary S2 and S3 Figs.**

Supplement: S15 Fig — (PDF) [file pone.0304183.s015.pdf]

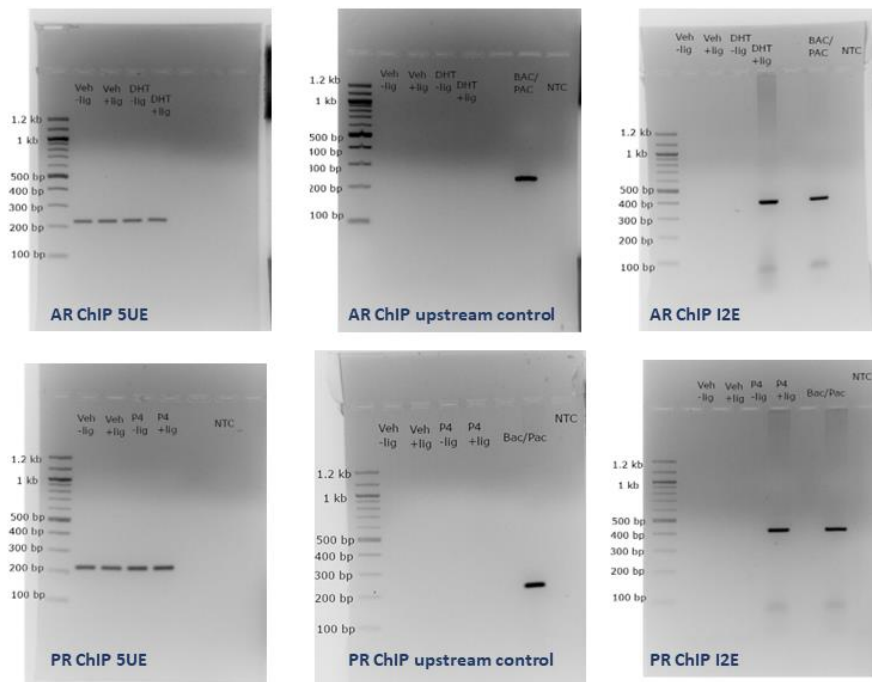

**S16 Fig. Uncropped gel images for data presented in S7 Fig.**

Supplement: S16 Fig — (PDF) [file pone.0304183.s016.pdf]
