# Supplementary material for: Mechanistic insights into steroid hormone-mediated regulation of the androgen receptor gene
Source: PLoS One. 2024 Aug 1;19(8):e0304183. doi: 10.1371/journal.pone.0304183 (PMC11293711; doi:10.1371/journal.pone.0304183)
Supplement: S3 Fig — (A) qRT-PCR of RNA samples harvested from VCaP cells treated with various ERβ ligands in the presence or absence of pDC315-ERβ-CFP. GAPDH used as an endogenous control for data normalisation. Error bars show ± standard error of the mean (3 biological replicates). ** = p < 0.01 (ANOVA with Tukey’s Post-Hoc test). (B) Anti-AR and Anti-PSA immunoblots of protein extracts taken from VCaP cells treated with various ERβ ligands in the presence or absence of pDC315-ERβ-CFP. β-Actin shown as a loading control. Blots are representative of 3 biological replicates. E2 = 17β-oestradiol. DPN = diarylpropionitrile. (PDF) [file pone.0304183.s003.pdf]

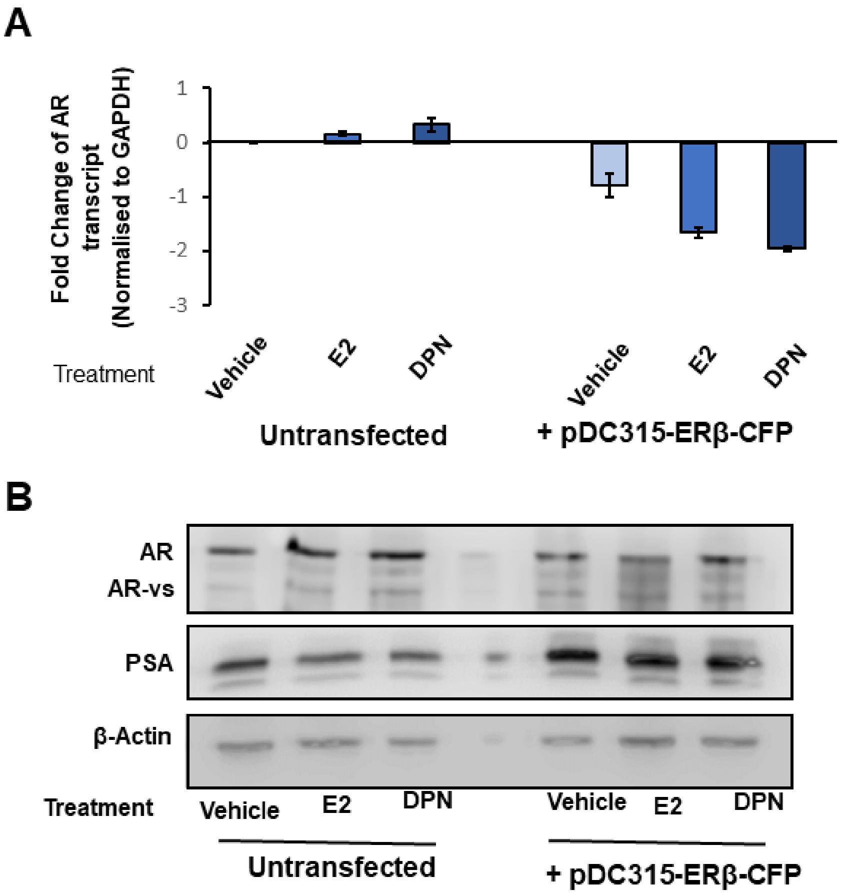


**S3 Fig. Transfection with estrogen receptor β affects the AR pathway in the absence of hormone.**
