# Supplementary material for: Mechanistic insights into steroid hormone-mediated regulation of the androgen receptor gene
Source: PLoS One. 2024 Aug 1;19(8):e0304183. doi: 10.1371/journal.pone.0304183 (PMC11293711; doi:10.1371/journal.pone.0304183)
Supplement: S7 Fig — Example qPCR 5-fold dilution series data for primers against (A) the AR 5’ UTR and (B) the AR Intron 2 element. Dilution series were carried out using Input ChIP samples. Ct value (average of 2 technical replicates) is plotted against dilution level across the dilution series. In both cases, this dynamic range covers experimental Ct values. qPCR efficiencies are shown as calculated by the formula efficiency = −1 + 10(−1/slope) to the nearest integer. These efficiencies fall within the 90%-110% acceptable range as outlined within the MIQE guidelines (Bustin et al. The MIQE guidelines: minimum information for publication of quantitative real-time PCR experiments. Clin. Chem. 2009; 55, 611–622). (PDF) [file pone.0304183.s007.pdf]

Primer Efficiency

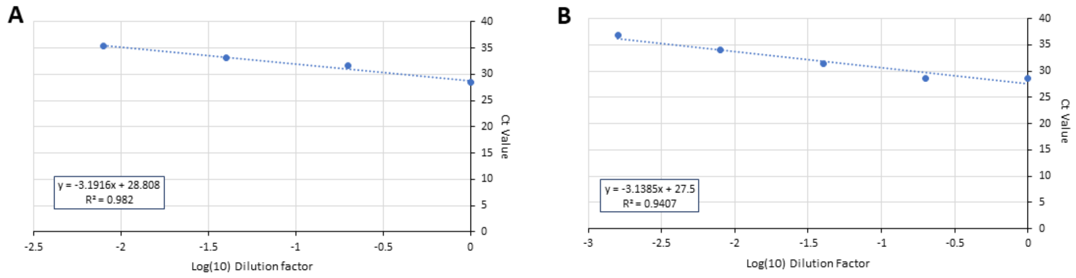

Efficiency = 106%

Efficiency = 108%

S7 Fig. Primer efficiencies for putative AR repressor sequences.
