# Supplementary material for: Mechanistic insights into steroid hormone-mediated regulation of the androgen receptor gene
Source: PLoS One. 2024 Aug 1;19(8):e0304183. doi: 10.1371/journal.pone.0304183 (PMC11293711; doi:10.1371/journal.pone.0304183)
Supplement: S12 Fig — VCaP cells were treated with increasing concentrations of either SAHA (A) or sodium butyrate (B). RNA was harvested 24 hours after treatment. In all cases, GAPDH used as an endogenous control. (PDF) [file pone.0304183.s012.pdf]

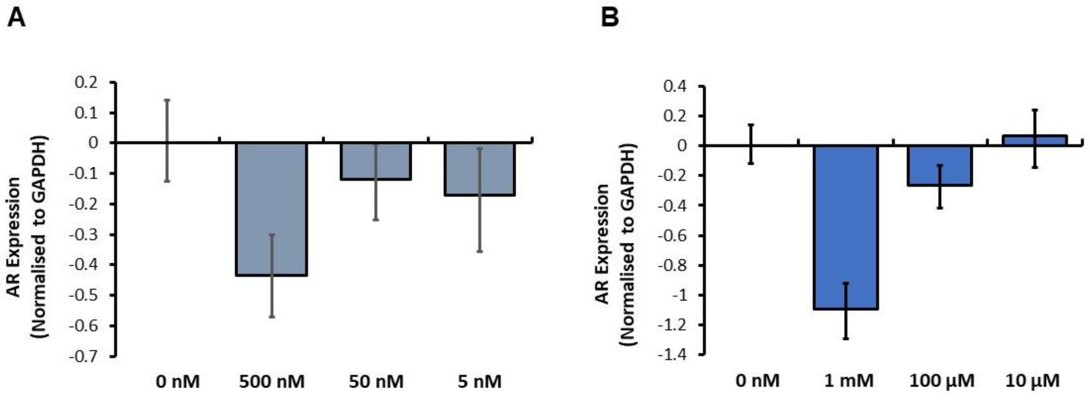

S12 Fig. Titration of HDAC inhibitors. VCaP cells were treated with increasing concentrations of either SAHA (A) or sodium butyrate (B). RNA was harvested 24 hours after treatment. In all cases, GAPDH used as an endogenous control.
