# Supplementary material for: Mechanistic insights into steroid hormone-mediated regulation of the androgen receptor gene
Source: PLoS One. 2024 Aug 1;19(8):e0304183. doi: 10.1371/journal.pone.0304183 (PMC11293711; doi:10.1371/journal.pone.0304183)
Supplement: S1 Table — (PDF) [file pone.0304183.s017.pdf]

S1 Table. A List of antibodies used in this study.

Target	Antibody	Species	Supplier
(C, G, Y) FP tag	AB121	Rabbit	Evrogen
AR (Immunoprecipitation)	PG-21	Rabbit	Millipore
AR (Western Blot)	AR 441	Mouse	Santa Cruz
ERα	D8H8	Rabbit	Cell Signalling
GR	ab2768	Rabbit	Abcam
PR (A and B)	D8Q2J	Rabbit	Cell Signalling
H3 acetyl (K9, K14, K18, K23, K27)	ab47915	Rabbit	Abcam
H3K4me2	ab7766	Rabbit	Abcam
H3K4me3	ab8580	Rabbit	Abcam
PSA	EP1588Y	Rabbit	NOVUS
β- actin	D6A8	Rabbit	Cell Signalling
Mouse Ig-HRP	A4416	Goat	Sigma
Rabbit Ig-HRP	A6154	Goat	Sigma

HRP = Horseradish peroxidase

FP = fluorescence protein
