# Supplementary material for: Mechanistic insights into steroid hormone-mediated regulation of the androgen receptor gene
Source: PLoS One. 2024 Aug 1;19(8):e0304183. doi: 10.1371/journal.pone.0304183 (PMC11293711; doi:10.1371/journal.pone.0304183)
Supplement: S2 Table — (PDF) [file pone.0304183.s018.pdf]

S2 Table. List of primers used in qPCR reactions.

Primer Name	Sequence (5' to 3')
AR cDNA (F)	GCCTTGCTCTCTAGCCTCAA
AR cDNA (R)	GGTCGTCCACGTGTAAGTTG
GAPDH (F)	AGCCACATCGCTCAGACAC
GAPDH (R)	GCCCAATACGACCAAATCC
AR Exon 3 (F)	AACAGAAGTACCTGTGCGCC
AR Cryptic Exon 3 (R)	TCAGGGTCTGGTCATTTTGA
AR Cryptic Exon 5 (R)	GCAAATGTCTCCAAAAAGCAGC
Genomic DNA Targets	
AR 5' UTR (F)	CGGAGAGAACCCTCTGTTTTCC
AR 5' UTR (R)	TCCTCCACCTTCCAAATTCAGTGT
AR Intron 2 (F)	CCATCATGTGCATTATGTGC
AR Intron 2 (R)	GGGACACTGATATGCACCAA
ChIP-Loop Intron 2	GGAAGAAATGCAAGTGAACCCTCATTGAACTC
ChIP-Loop TSS	CCGACTCGCAAACTGTTGCATTTGCTC
ChIP-Loop Upstream Control	GGGAGACCTCACCACCCTAAAAGAAAGAA

F= Forward

R= Reverse
