# Supplementary material for: Mechanistic insights into steroid hormone-mediated regulation of the androgen receptor gene
Source: PLoS One. 2024 Aug 1;19(8):e0304183. doi: 10.1371/journal.pone.0304183 (PMC11293711; doi:10.1371/journal.pone.0304183)
Supplement: Attachment — Submitted filename: Reply to Reviewers R1 and R2.pdf [file pone.0304183.s021.pdf]

Response to Reviewers-R1

Reviewer 1

1. We agree with the reviewer that it is important to consider the impact of exogenous expression of proteins. However, it is important to note that all the experiments reported with exogenous expression of the progesterone receptor (PR) and endogenous levels of androgen receptor (AR) are plus/ minus hormone. In the ChIP experiments (Figure 3) for example there is clear recruitment of AR and PR after hormone treatment which is not seen with a non-specific IgG control. We also note that VCaP cells are widely reported to over express the endogenous AR and a comparison of the levels of AR (Figure 2, Supplementary Figure S1) with exogenous PR (A/B) (Supplementary Figure S2) are not so different, relative to the house keeping control. In contrast we do see significant over expression of the ERα (Supplementary Figure S2), but no significant impact of this receptor in the absence or presence of hormone. Furthermore, we have redrawn the data in Supplementary Figure S4 showing that there is no effect of P4 in the absence of PR. Therefore, we conclude our results cannot simply be explained because of receptor overexpression and collectively, we can be confident that we are seeing effects of the hormone activated PRB in our experiments.
2. We apologise for this oversight, the number of independent blots was n=3, which is now included in the figure legend. We now also include the full blots as supplementary material (Supplementary Figure S11).
3. We thank the reviewer for this comment and would like to take this opportunity to state that our experiments were designed and analysed carefully and apologise that this did not come across in the text. We have now corrected the text throughout for clarity and to demonstrate careful analysis and interpretation of the results as suggested.
4. As point 3 above.
5. As point 3 above.
6. Agreed and this has been done.
7. We agree this was an oversight and we have now included the additional references [13 to 16] to a far upstream enhancer element. However, this needs to be placed in the context of the present study which was addressing the mechanism of steroid receptor downregulation of the AR mRNA. The upstream enhancer is interesting as it appears to have features of a switch regulating AR during development and reactivated during progression to therapy resistant prostate cancer. However, there is no evidence it is involved in the AR (our work and others) or PR-negative regulation (present study) of receptor mRNA. We have also revised the text of this paragraph to more accurately reflect the work cited (lines 85 to 101).
8. We are happy to acknowledge limitations of the present study (Discussion). However, the role of PR in the regulation of the AR gene is supported by other studies in the literature and our own analysis of publicly available data sets. Our work contributes to this body of evidence by providing mechanistic insights, which can be further tested, for example in a cell model with endogenous levels of both AR and PR (line788-789).
9. Corrected
10. The text has been proofread and corrected where necessary.
11. Text has been corrected as suggested.

Reviewer 2

1. We agree with the reviewer this is an important point and indeed informed our choice of VCaP and PC3 (AR negative) cells as our principal cell models. LNCaP cells which are widely used due to being hormone-sensitivity and ease of growing, have mutated AR (T878A), which permits activation by progesterone amongst other ligands. Similarly, 22RV1 cells have mutated AR. To address this concern, we have revised Supplementary Figure 4, to indicate

that there was no effect of P4 alone in the absence of PR transfection, eliminating the possible effects of P4 on AR. Unfortunately, we did not have a PR antagonist in these studies.

2. We thank the reviewer for this comment. We have analysed patient data as suggested- included here for review purposes only. We are planning a separate manuscript focusing on the role of PR in prostate cancer, including our own IHC data and patient data sets. However as shown in the figure below (TCGA data set), there is a clear decrease in PR levels, which contrast with increased AR expression and is consistent with our model that loss of PR repression will lead to increased levels of AR.

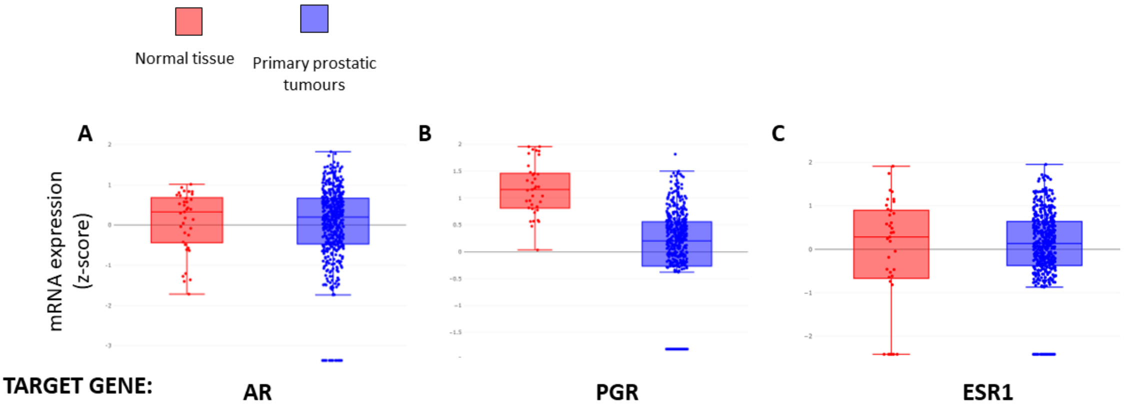

F

Figure Legend- Expression of Steroid Hormone Receptor genes in primary prostate tumours. Expression of **A)** AR; **B)** PGR; **C)** ESR1 were analysed in the 333 primary prostate cancer samples available through the TCGA Research Network (https://www.cancer.gov/tcga). This analysis was carried out using PIXdb (Marzec et al., 2021). Relative expression of these genes in normal tissue (red) and prostatic tumours (blue) are shown compared to the z-score for that RNA. Z-score of a gene is calculated by comparing its expression level in a given sample to the expression level of that gene across all samples.

Our aim with the present study- manuscript - was to investigate the underpinning mechanism of steroid receptor regulation of the AR gene.

3. No there was no effect of P4 in untransfected cells, and we have clarified this with revision of Supplementary Figure S4 (see also comments to Point 1 above).
4. We agree with the reviewer this is puzzling. The assumption is cross-reactivity of the antibody and have made a comment to this effect.
5. Although this manuscript was not focussing on prostate cancer specifically, we agree that the regulation of splice variants is a very important and an interesting aspect of AR biology. We have now included data for both 22RV1 and VCaP cells showing hormone (DHT or P4) regulation of AR-Vs (new Supplementary Figure S5) (text lines 409 to 417).
6. We agree this would be interesting to include, but a single concentration of hormone was chosen for consistency across the multiple experimental methods used.
7. In our VCaP cells, change in AR RNA correlated with changes in receptor protein-levels, with DHT treatment significantly reducing both transcript and protein levels. Whilst DHT treatment reduced AR transcript to only one quarter of control levels, the effect on AR protein was much less pronounced, with less than a 50% reduction noted. This has been reported widely previously by us and others: while DHT-mediated repression of the AR protein has also been noted in VCaP cells previously. However, as stated by the reviewer, investigation in other prostate cancer cell lines have noted DHT-mediated increase in total AR protein in LNCaP, 22Rv1 and LAPC4 cells (Cai et al., 2011; Krongrad et al., 1991). The reason

for this difference is unclear but maybe related to a difference in baseline AR expression: VCaP cells contain an amplification of the AR gene, in fact possessing almost thirty copies of the AR gene, resulting in vastly increased AR RNA and protein levels (Korenchuk et al., 2001; Makkonen and Palvimo, 2011). As such, it is possible that the effect of DHT on AR stabilisation is somewhat diluted versus the total pool of available AR, and so the effects of reduced protein production can be noted. Thus, in LNCaP androgen reduces mRNA but stabilises protein, while in VCaP androgens decrease both mRNA and protein. To the best of our knowledge the mechanistic basis for this is unknown. However, we have added a sentence to clarify this in the text (lines 424-427).

8. We appreciate the concern raised and have added a sentence to highlight this point (lines 693-695).
9. Again, we appreciate the concern raised and include additional supporting data to show that the concentrations of SAHA or sodium butyrate used do not impact on control AR mRNA (Supplementary Figure S10).
10. Due to mutations in the AR leading to promiscuous hormone binding we were limited in the choice of prostate cell models. The reviewer makes a good suggestion, and a follow-up study might address this in breast cancer model (MCF-7) where both AR and PR are endogenously expressed. As discussed above neither the over expression of PR alone or P4 alone is sufficient to regulate AR gene expression. However, we have now included a statement regarding the value of repeating the study with cells expressing AR and PR endogenously (lines 788-789).
11. This is an important point and we have now clarified the analysis and statistical tests used throughout in the revised manuscript and updated the relevant part of the methods.
12. We agree and have expanded the text of the Introduction to acknowledge this work on a distal enhancer and provide a fuller description of previous work [references 13 to 16] (lines 85 to 101).

Response to Reviewers' Comments R2

Reviewer 1

We have carefully read over the revised manuscript and have corrected all typos found.

Reviewer 2

We thank the reviewer for their additional comments and suggestions.

As we explained in out earlier response to the concern on the use exogenous expression of the PR, there is clear methodological rationale and the effects observed are regulated by hormone, not simply the presence of the receptor protein. We evidence this by the data in Supplementary Figure S4 and throughout the manuscript. We also acknowledged the limitations of this approach in our earlier revision of the manuscript and the importance of further studies in cells expressing endogenous AR and PR. However, these experiments are out with the scope of the present study. However, it is also important to stress the use of exogenous expression is both a widely used and accepted methodology in dissecting molecular signalling pathways.

It is precisely because the 3-keto receptors (AR, GR, PR and MR) share homology in their DNA binding domains and in the DNA recognition sequences of hormone response elements that we were excited

to report the detailed molecular programme of work described in this manuscript. Indeed, we reference this point in the Introduction (text lines 108 to 114).

1. Interpreting the ChIP studies. We have now included additional supporting data demonstrating that the primer sets target the two genomic regions of the AR gene with comparable efficiencies (Supplementary Figure S7, text lines 451 to 452). Therefore, collectively we would argue that our interpretation of the evidence presented is consistent with preferential recruitment of both the AR and PR-B at the Intronic site. We accept that other interpretations maybe possible, that is the nature of science.
2. RT-qPCR was not done on either the ER or PR mRNA transcripts, which is why there is no information on primers. All primers used in qPCR are listed in Table 2 and we have now included a Supplementary Figure (S5) showing the specificity of the AR primers used and a single amplified product of 103 bp (text lines 377 to 382).
3. We are grateful to the reviewer for drawing our attention to this error. We have now included the correct gel images in Figure 2A (middle and right panels). It is important to state this does not affect the quantification shown in Part B and subsequent conclusions.
4. This is an interesting point; however, the main conclusions of this study were the regulation of the *AR* gene by progesterone acting through PR-B protein. In this context it should be noted that we do see an impact on the AR target gene PSA (KLK3) protein levels in response to P4/ PR-B, most likely as a consequence of downregulated AR expression (Figure 2C and text lines 419 to 421).
