# Supplementary material for: Mechanistic insights into steroid hormone-mediated regulation of the androgen receptor gene
Source: PLoS One. 2024 Aug 1;19(8):e0304183. doi: 10.1371/journal.pone.0304183 (PMC11293711; doi:10.1371/journal.pone.0304183)
Supplement: Attachment — Submitted filename: Reply to reviewers R2.docx [file pone.0304183.s020.docx]

**Response to Reviewers’ Comments R2**

**Reviewer 1**

We have carefully read over the revised manuscript and have corrected all typos found.

**Reviewer 2**

We thank the reviewer for their additional comments and suggestions.

As we explained in out earlier response to the concern on the use exogenous expression of the PR, there is clear methodological rationale and the effects observed are regulated by hormone, not simply the presence of the receptor protein. We evidence this by the data in Supplementary Figure S4 and throughout the manuscript. We also acknowledged the limitations of this approach in our earlier revision of the manuscript and the importance of further studies in cells expressing endogenous AR and PR. However, these experiments are out with the scope of the present study. However, it is also important to stress the use of exogenous expression is both a widely used and accepted methodology in dissecting molecular signalling pathways.

It is precisely because the 3-keto receptors (AR, GR, PR and MR) share homology in their DNA binding domains and in the DNA recognition sequences of hormone response elements that we were excited to report the detailed molecular programme of work described in this manuscript. Indeed, we reference this point in the Introduction (text lines 108 to 114).

1. Interpreting the ChIP studies. We have now included additional supporting data demonstrating that the primer sets target the two genomic regions of the AR gene with comparable efficiencies (Supplementary Figure S7, text lines 451 to 452). Therefore, collectively we would argue that our interpretation of the evidence presented is consistent with preferential recruitment of both the AR and PR-B at the Intronic site. We accept that other interpretations maybe possible, that is the nature of science.
2. RT-qPCR was not done on either the ER or PR mRNA transcripts, which is why there is no information on primers. All primers used in qPCR are listed in Table 2 and we have now included a Supplementary Figure (S5) showing the specificity of the AR primers used and a single amplified product of 103 bp (text lines 377 to 382).
3. We are grateful to the reviewer for drawing our attention to this error. We have now included the correct gel images in Figure 2A (middle and right panels). It is important to state this does not affect the quantification shown in Part B and subsequent conclusions.
4. This is an interesting point; however, the main conclusions of this study were the regulation of the *AR* gene by progesterone acting through PR-B protein. In this context it should be noted that we do see an impact on the AR target gene PSA (KLK3) protein levels in response to P4/ PR-B, most likely as a consequence of downregulated AR expression (Figure 2C and text lines 419 to 421).
